# Lung inflammation after repeated exposure to LPS and glyphosate in female mice

**DOI:** 10.1038/s41598-026-58018-8

**Published:** 2026-06-17

**Authors:** Kaitlin Merkowsky, Shelley Kirychuk, Gurpreet K. Aulakh, Brooke Thompson, Upkardeep S. Pandher, David Schneberger, Baljit Singh, Farangis Foroughi, Dean Chapman

**Affiliations:** 1 Canadian Centre for Rural and Agricultural Health, University of Saskatchewan, 104 Clinic Place, P.O. Box 23, Saskatoon, Saskatchewan, S7N 2Z4 Canada; 2 Department of Medicine, College of Medicine, University of Saskatchewan, 104 Clinic Place, P.O. Box 23, Saskatoon, Saskatchewan, S7N 2Z4 Canada; 3 Small Animal Clinical Sciences, Western College of Veterinary Medicine, University of Saskatchewan, 52 Campus Drive, Saskatoon, Saskatchewan, S7N 5B4 Canada; 4 Department of Veterinary Biomedical Sciences, Western College of Veterinary Medicine, University of Saskatchewan, 52 Campus Drive, Saskatoon, Saskatchewan, S7N 5B4 Canada; 5 Department of Physics and Engineering Physics, College of Arts and Science, University of Saskatchewan, 116 Science Place, S7N 5E2 Saskatoon, Saskatchewan, Canada; 6 Department of Computer and Electrical Engineering, Mid Sweden University, Sundsvall, Sweden; 7 Department Anatomy, Physiology & Pharmacology, College of Medicine, University of Saskatchewan, 107 Wiggins Road, Saskatoon, Saskatchewan, S7N 5E5 Canada

**Keywords:** Diseases, Environmental sciences, Medical research

## Abstract

Agricultural work involves exposure to airborne pollutants including dust and pesticides that can cause respiratory effects, yet little is known about these impacts in females. The inflammatory effects of inhaled glyphosate, alone or in combination with common agricultural exposures like lipopolysaccharide (LPS), remain unclear. The objective was to evaluate the inflammatory potential of single and combined exposures to glyphosate and LPS in female mice using physiological and structural measures, and synchrotron imaging. C57BL/6 female mice (*n* = 20) were intranasally treated with glyphosate (GLY), LPS, LPS + glyphosate (LG), or HBSS (CTL) for 5 days. On day 5, an additional group of mice were transported to the Canadian Light Source synchrotron (CLS) for multiple image x-radiography (MIR) to assess lung injury. Following treatment, mice were euthanized and bronchoalveolar lavage fluid (BAL) and lung tissue were collected. Mice exposed to LG had significantly higher airway restriction; expression of pro-inflammatory cytokines/chemokines TNF-α, KC, IL-6, MCP-1, and MIP-2; levels of myeloperoxidase expression; greater recruitment of cells into the alveolar regions, disruption to the bronchial epithelium in the lungs, and compromised lung air-tissue interfaces in the MIR images compared to other treatment groups. It is likely that inflammatory adaptation is already occurring in the female mice by five days of exposure. These results reveal that female mice exposed to LG displayed physiological, structural, and lung injury effect that were different from mice exposed to LPS and GLY alone.

## Background

Workers in the agriculture industry are at risk for developing deleterious respiratory effects from workplace exposures including grain dust and pesticides^1^. Workplace exposures to dust containing lipopolysaccharide (LPS) and pesticides are common, and these respiratory exposures can often occur simultaneously. It is unknown if there is a differential respiratory effect when exposures are combined compared to an individual exposure. This is the first study to evaluate the potential additive and/or synergistic inflammatory effects of exposure to a combination of LPS and glyphosate in female mice.

Lipopolysaccharides (LPS), also known as endotoxins, are a class of substances that are a major component of the outer surface membrane of Gram-negative bacteria^2,3^. LPS are also a component of agricultural dust. A low dose of LPS is a standard method to evaluate the innate immune response during inflammation^4^ or to model acute lung injury^5^ that induces a well characterized increase in cytokines. For example, TNF-α, IL-1ß, IL-6, and IL-10 may be measured along with other indicators of inflammation such as levels of leukocytes, C-reactive protein, heat shock proteins or LPS-binding proteins^5^. Some studies indicate LPS may act as an adjuvant and enhance the immune response when the immune system is exposed to other toxicants such as pesticides^5,6^. There is mounting evidence that exposures to pesticides, in combination with LPS, produces an inflammatory response that is significantly different compared to the response measured from exposure to the pesticide alone^7^.

Worldwide, glyphosate-based herbicides, are the most commonly used herbicides^8^, in both industrialized and developing countries^9^. Glyphosate has been shown to have a significant association with increased rhinitis episodes amongst pesticide applicators^10^.

There is limited data available on the in vivo inflammatory effects of glyphosate alone or in combination with LPS. Kumar et al. used aerosol samplers (Button Inhalable Aerosol Sampler, SKC Inc., Eighty-Four, PA) to collect glyphosate samples over a 24-hour period during spray season at 3 farms in Ohio^11^. The glyphosate residues were extracted from suspensions eluted from the filters, and airborne concentration of glyphosate was found to be 22.59 ng/m^3^^11^. The same researchers also extracted endotoxin from the collected filters and levels were determined to be at an average airborne concentration of 4.87 EU/m^3^. For intranasal instillation of glyphosate, female mice were given either aerosolized glyphosate from one of the farm samplers used (8.66 mg/ml in 30 ml of PBS) daily for 7 days, or reagent grade glyphosate at 100 ng, 1 mg or 10 mg for 7 days^11^. The results of this study found female mice exposed to either glyphosate-rich air samples or reagent grade glyphosate experienced an increase in cell count in lung and bronchial alveolar lavage, an increase in eosinophil and neutrophils, and increased production of IL-33, TSLP, IL-13 and IL-5^11^.

Previous studies looked at a glyphosate exposure in combination with LPS over a 1, 5, or 10-day period in male mice to measure the respiratory inflammatory response^7^. A 5-day intranasal exposure at an agriculturally relevant concentration of glyphosate (1 µg/40 µL)^11^ plus LPS resulted in a significantly higher number of neutrophils and increased expression of TNF- α, IL-6, and KC in bronchoalveolar lavage fluid, and higher myeloperoxidase levels in lung tissue compared to the control, LPS, and GLY treated animals. The LG treated animals also had significantly higher expression of MIP-2 compared to the control and GLY animals, and higher MCP-1 in bronchoalveolar lavage fluid compared to the control animals^7^. Additionally, lung immunohistochemistry showed repeated exposure to glyphosate results in leukocyte infiltration and increased expression of adhesion molecules ICAM-1 and VCAM-1 in the peribronchiolar, perivascular, and alveolar regions^12,13^ indicating capacity for inflammation to be induced from a co-exposure of LPS plus glyphosate.

Multiple-image radiography (MIR) is an X-ray phase-based imaging method utilizing monochromatic synchrotron x-rays that generates contrast from the air-tissue interfaces of the lung^14,15^. This method is a variation of diffraction enhanced imaging (DEI) and can be used to quantify the optical properties of acute lung injury following LPS instillation^14^. Due to the high number of air-tissue interfaces present in the lungs, they are an excellent candidate for MIR imaging^14^ where it is possible for the investigator to make assessments of subtle structural changes in the lungs following exposure to agents such as LPS and/or glyphosate and provide a mechanism to correlate those with physiological changes. The number of air-tissue boundaries (alveoli) present in the lung are proportional to the MIR parameter ultra-small-angle-x-ray scatter (USAXS) which may also be referred to as “width”^16^. In a study utilizing MIR to evaluate longitudinal changes in the lungs following LPS exposure in male mice, it was found that x-ray refraction and scatter were reduced following LPS exposure which represented an increase in fluid buildup, or higher levels of edema in the lungs^17^.

Much of the current literature to date including the studies cited above focuses on exposure effects of male mice. This paper addresses the gap in research on the female response to common agricultural exposures of LPS and glyphosate. Further, to the best of our knowledge this is the first study to use Multiple Image Radiography (MIR) to evaluate the effects of multiple short-term agriculture respiratory exposures, and in a female mouse population. This study is the first to evaluate the effects of both individual exposures and combined exposures to LPS and glyphosate through physiological markers, structural measures, and lung injury assessments in female mice.

## Materials and methods

### Animals

C57BL/6 female mice (Charles River Laboratory, Quebec, Canada) aged 8–10 weeks were used for this study. The mice were housed in ventilated cages in the Laboratory Animal Services Unit (LASU) at the University of Saskatchewan where commercial rodent chow and water was available *ad libitum*. The animals were acclimatized to the whole-body plethysmography (WBP) chambers (Buxco FinePointe Whole Body Plethysmography 4-site system; Data Sciences International, Minneapolis, MN) for 20 min per day at least twice prior to the start of treatments. Experiment protocols were approved by the Animal Ethics Research Board of the University of Saskatchewan (AUP 20190017). All animal work was performed in accordance with the Canadian Council on Animal Care guidelines.

### Study design for physiological measures and structural changes

Twenty mice were randomly divided into 4 groups (*n* = 5/group). Nasal instillation was used to administer either LPS, glyphosate (GLY), LPS + glyphosate (LG), or Hank’s Balanced Salt Solution (HBSS; CTL). The doses of LPS and glyphosate were determined as previously described by Pandher et al.^7^, and in short, based on Guyton’s formula^18^, the minute volume of a mouse with average weight 20 g^18^, airborne endotoxin levels commonly found in agriculture^19^, and airborne glyphosate levels commonly found in agriculture^11^. The control group received 40 µL of HBSS (Life Technologies, Grand Island, NY, without calcium, pH 7.4). The LPS group received LPS in HBSS (0.5 µg/40µL) (1 mg/mL; *E. coli* serotype 0111:B4, Sigma, St Louis, MO). The GLY group received GLY in HBSS (1 µg/40 µL) (0.85 M; analytical grade PESTANAL standard, Sigma, St Louis, MO). The LG group received both LPS and GLY in HBSS (0.5 µg LPS + 1 µg GLY/40 µL). Mice were lightly anesthetized using isoflurane and treated intranasally with 40 µL of the treatment solution. Treatments were given daily for 5 days to mimic a typical work week. Following each exposure, mice were allowed to recover from anesthesia in a separate container before being placed back in their respective cages. After exposure on the 5th day, mice were placed back in the cages for 4 h. Following the four-hour period, mice were placed in the WBP chambers for 20 min to collect respiratory data. Once WBP analysis was complete, mice were euthanized via carbon dioxide overdose (0.6 to 0.8 L/min) under light anesthesia using isoflurane (2.5% isoflurane delivered in O_2_ at 2 L/min), followed by cervical dislocation; and blood, BAL, and lung tissue were collected and analyzed.

## Sample collection and analysis

### Whole body plethysmography

Four hours after treatment on day 5, mice were placed into whole body plethysmography chambers to monitor the respiratory changes according to the manufacturer’s protocol. Mice were placed into calibrated chambers and respiratory measures (enhanced pause {PenH}, average frequency of breath, minute volume, and peak expiratory volume) were recorded every 2 s for 20 min; these results were averaged per animal. Animals were continuously monitored for any indications of pain or distress. Data collection was done using FinePointe software (Data Sciences International, Wilmington, NC).

### Hormone analysis

The blood was collected from mice via cardiac puncture. Whole blood with EDTA was stored in -80 °C until further analysis. Blood samples (*n* = 4/treatment group) were sent to Eve Technologies (Calgary, AB) for quantification of estradiol, testosterone, cortisol, progesterone, triiodothyronine (T3), and thyroxine (T4) using the steroid/thyroid hormone 6-plex discovery assay.

### Bronchoalveolar lavage

The bronchoalveolar lavage (BAL) fluid was collected by lavaging the lungs three times with 0.5 mL cold HBSS. BAL fluid was centrifuged at 1000 x g for 10 min at 4 °C and the supernatant was stored at -80 °C for future analysis. The cell pellet was resuspended in HBSS and kept on ice. Total leukocyte counts (TLC) were measured using a hemocytometer and expressed as an absolute number. Total protein concentration in BAL fluid was determined using the Pierce BCA Protein Assay Kit (ThermoFisher Scientific, Waltham, MA). The plate was read at 562 nm on a BioTek SynergyHT plate reader (BioTek, Winooski, VT) and Gen5 2.09 Software (BioTek, Winookski, VT).

### Lung tissue collection

Following BAL fluid collection, the right lung was tied at the primary bronchus, removed, flash-frozen in liquid nitrogen and stored in -80 °C for mRNA and protein analysis. The left lung was inflated with 200 µL of 4% paraformaldehyde (PFA), removed and fixed in 4% PFA for 16 h at 4 °C followed by two overnight washes with 70% ethanol. The fixed tissues were processed through a series of alcohols in an Intelsint RVG/1 Histology Vacuum tissue processor (Intelsint; Turin, Italy), followed by embedding in paraffin using a Tissue Tek II tissue embedder (Sakura Finetek; Nagano, Japan).

### Eosinophil peroxidase (EPO), and myeloperoxidase levels (MPO)

Lung homogenates were prepared using 2 mm Zirconia beads (BioSpec, Bartlesville, OK) in tubes containing 500 µL RIPA Lysis Buffer containing 1X Halt Protease and Phosphatase Inhibitor Cocktail (ThermoFisher Scientific, Waltham, MA) and homogenized using a BioSpec Mini-Beadbeater-24 homogenizer (BioSpec, Bartlesville, OK) twice for 2 min, with cooling on ice for 5 min in between rounds. Total Protein concentration of the lung homogenates was determined using the Pierce BCA Protein Assay (ThermoFisher Scientific, Waltham, MA) according to manufacturers’ instructions for the microplate assay. Myeloperoxidase (MPO) was quantified using a Mouse Myeloperoxidase DuoSet ELISA (R&D Systems, Minneapolis, MN). Eosinophil Peroxidase (EPO) was quantified using a Mouse Eosinophil Peroxidase/EPX ELISA assay (Lifespan Biosciences, Seattle, WA). Plates for MPO and EPO quantification were read at 450 nm on a BioTek SynergyHT plate reader (BioTek, Winooski, VT) and Gen5 2.09 Software (BioTek, Winooski, VT).

### Lung histology

Sections of 5 μm thickness were cut from the embedded lung tissue using an Microm 350 S microtome (Microm, Germany). Tissue sections were stained with Hematoxylin and Eosin according to the Histology Core Facility’s protocol^20^ and coverslipped using Surgipath MM24 Mounting Media (Leica Biosystems, Richmond, IL USA). These slides were imaged on an Aperio CS2 virtual microscopy system (Leica Biosystems, Concord ON Canada) as well as a bright field microscope equipped with an Infinity 5–5 Microscope camera (Teledyne Lumenera, ON Canada) at 10, 40 and 100x magnification.

### Immunohistochemistry

Lung sections were incubated with primary antibodies followed by the appropriate secondary antibody. Briefly, lung sections were rehydrated through a series of xylene and descending grades of alcohol. Lung sections were covered with 0.5% hydrogen peroxide in methanol for 20 min to quench tissue peroxidase activity. The sections were covered with 2 mg/mL of pepsin in 0.01 N hydrochloric acid (HCl) for 45 min to unmask antigens. Next the sections were covered in 1% bovine serum albumin (BSA) for 30 min to block non-specific binding sites. The sections were incubated overnight at 4°˚C with the primary antibody in 1X TBS + 1% BSA. The following primary antibodies and concentrations were used: ICAM-1 (dilution 1:100; rabbit monoclonal anti-mouse ICAM-1, ab79707, Abcam Inc., ON Canada). Following overnight incubation, the sections were covered with anti-rabbit secondary antibody (Agilent Dako, Mississauga, ON). Sections were covered with NovaRed solution (Vector laboratories, Burlington, ON Canada) for 1–5 min and then counter-stained with methyl green nuclear stain for 5 s. Sections were coverslipped using Surgipath MM24 Mounting Media (Leica Biosystems, Richmond, IL, USA). Von Willebrand Factor (dilution 1:200; rabbit monoclonal anti-mouse vWF, ThermoFisher Scientific, Waltham, MA, USA) was used as a positive control. Additionally, controls of staining without primary antibody, secondary antibody, or both, were completed.

### Multiplex ELISA

Quantification of Tumor Necrosis Factor - alpha (TNF-α), Interleukin (IL)-6, keratinocyte chemoattractant (KC), Monocyte chemoattractant protein − 1 (MCP-1), macrophage inflammatory protein-2 (MIP-2), IL-1β IL-10, IL-13, IL-5, IL-4, IL-33 in BAL fluid was determined using a Custom ProcartaPlex Luminex Immunoassay (ThermoFisher Scientific, Waltham, MA) according to the manufacturer’s specification for magnetic bead-based ELISAs. Plates were read using a Bioplex 200 system (Bio-Rad, Mississauga, ON) and Bioplex Manager Software (Bio-Rad, Mississauga, ON).

### RNA purification, cDNA synthesis and Real-time PCR

RNA was extracted from lung tissue using Qiagen RNeasy Plus Mini Kit (Qiagen, Chatsworth, CA USA) according to manufacturer’s instructions for tissue extraction. The concentration of total RNA was quantified for each sample using a Take3 plate and BioTek Synergy HT plate reader (BioTek, Winooski, VT USA). cDNA was generated using iScript Reverse Transcription Supermix (Bio-Rad, Hercules, CA USA) using 0.5 µg mRNA according to the manufacturer’s instructions.

Real-time PCR reactions were carried out in duplicate in a CFX96 Touch Real-Time PCR Detection System (Bio-Rad, Hercules, CA USA). Expression of mouse TLR-2 (Mm00442346_m1), TLR-4 (Mm00445273_m1), HSPa1a (Mm01159846_s1) ICAM-1(Mm00516023_m1), and TNFAIP3 (Hs00234713_m1) (Life Technologies, Grand Island, NY USA) were assessed. The reaction was first incubated at 50 °C for 2 min, at 95 °C for 10 min, then 40 cycles of 15 s at 95 °C, then finally 1 min at 60 °C. To determine the relative quantification of each target gene, the ΔΔCt method was used.

### Statistical analysis

GraphPad Prism 9 (Graph-pad Software, San Diego, CA) was used to complete statistical analysis and preparation of graphs. Data are presented as mean + standard deviation (SD). For cytokine levels below the predicted limit of detection a value less than the lowest detected value, or a value equal to half the lowest level of quantification (LLOQ) was used. Statistical significance was determined using one-way ANOVA with a follow-up Tukey test for multiple comparisons. If the assumption of equal variance using the Brown’s Forsythe test was not met, a non-parametric Kruskal Wallis (KW) test was used. A *p-*value of ≤ 0.05 was considered significant.

### Multiple image radiography experiment methodology

Twelve mice were randomly divided into 4 groups (*n* = 3/group), as described above. Treatments were given daily for 5 days.Following treatment on day 5 day, mice were transported by van, in their cages, to the Canadian Light Source (CLS) at the University of Saskatchewan, Canada. The mice were anesthetized using and intraperitoneal (IP) injection of a combination of 100 mg/kg ketamine (Ketalar^®^, Pfizer, US) and 20 mg/kg xylazine (Rompun^®^, Bayer, US). Anesthesia was maintained with further 0.05–0.1 mL intraperitoneal injections with the same anesthetic combination using return of the pedal reflex as a guide to anesthetic depth. Mice were stationed on a plexi-glass holder and transferred to the imaging hutch. An infra-red heat lamp to maintain body temperature at 37 °C was used and body temperature was continuously monitored using a rectal probe. Eye lubrication gel was used to protect the corneas. Pulse oximetry was used to measure hemoglobin oxygen saturation (SpO_2_) by placing a probe on the right hind paw. The mice were maintained at normal body temperature and SpO_2_ were monitored throughout the study. The breathing rate was calculated by counting the number of diaphragmatic movements during one minute from the control room using a camera directed towards mice.

Mice were imaged at the Biomedical Imaging and Therapy - Bending Magnet (BMIT-BM) beamline of the CLS located at the University of Saskatchewan, Canada, using the protocol previously described by Aulakh et al.^14^. Briefly, all objects were positioned in hutch POE-2 of the BMIT beamline, 24 m downstream from the synchrotron source. The vertical and horizontal beam size is 4.0 mm and 250.0 mm, respectively. Si (2,2,0) planes were used in the double crystal monochromator to select 30 keV with a matching analyzer positioned between the mouse and the detector. A Photonic Science SCMOS detector (Photonic Science, UK), was placed 1.0 m downstream. This detector has a 25 × 25 μm pixel size with a field of view of approximately 75 mm (horizontal) x 50 mm (vertical). Due to the limited beam time, 3 mice were imaged in sequential vertical fields of view with approximately 12 milliGy/s surface dose per image. Each field of view with a MIR dataset contains 14 images with angular settings of the analyzer crystal and the acquisition time was 0.55 s/image.

Following image acquisition, mice were euthanized, and blood, BAL, and lung tissues were collected and analyzed as per the prior protocol.

### MIR image analysis

Fourteen object and flat images were acquired as the analyzer rotated at angles around the diffraction condition for the chosen energy i.e. 30 keV. In order to normalize the image data, the MIR images taken with a closed shutter or beam off (also called as the dark image) were subtracted from the object and flat field images. The 14 image sets were then parameterized by fits to a Gaussian function^21,22^.

This resulted in three Gaussian parameters; amplitude, center angle and width (USAXS). These fits were done with every pixel in the acquired object and flat field images. With these parameters, an intensity image could be created for the object and the matching flat. The difference between the object center angle and the flat center angle images results in the object’ refraction angle image. The scatter width (sometimes called an ultra-small angle x-ray scattering or USAXS image) is created by subtracting, in quadrature, the flat scatter width image from the object scatter width image. A combination of the amplitude and USAXS image can be used to compute a “transmission” image or a radiograph. Finally, for image sets where multiple exposures were taken to cover a larger vertical size of the lungs, the images were stitched together to form the final images.

The high contrast of lungs in the USAXS image was then used to define the outline of the right and left lungs and select them from the rest of the image. As USAXS squared parameter (µrad^2^) is directly proportional to thickness of scattering interfaces, this parameter was chosen to plot the “median USAXS parameter” across groups^23^.

## Results

### Whole body plethysmography

**Fig. 1:**
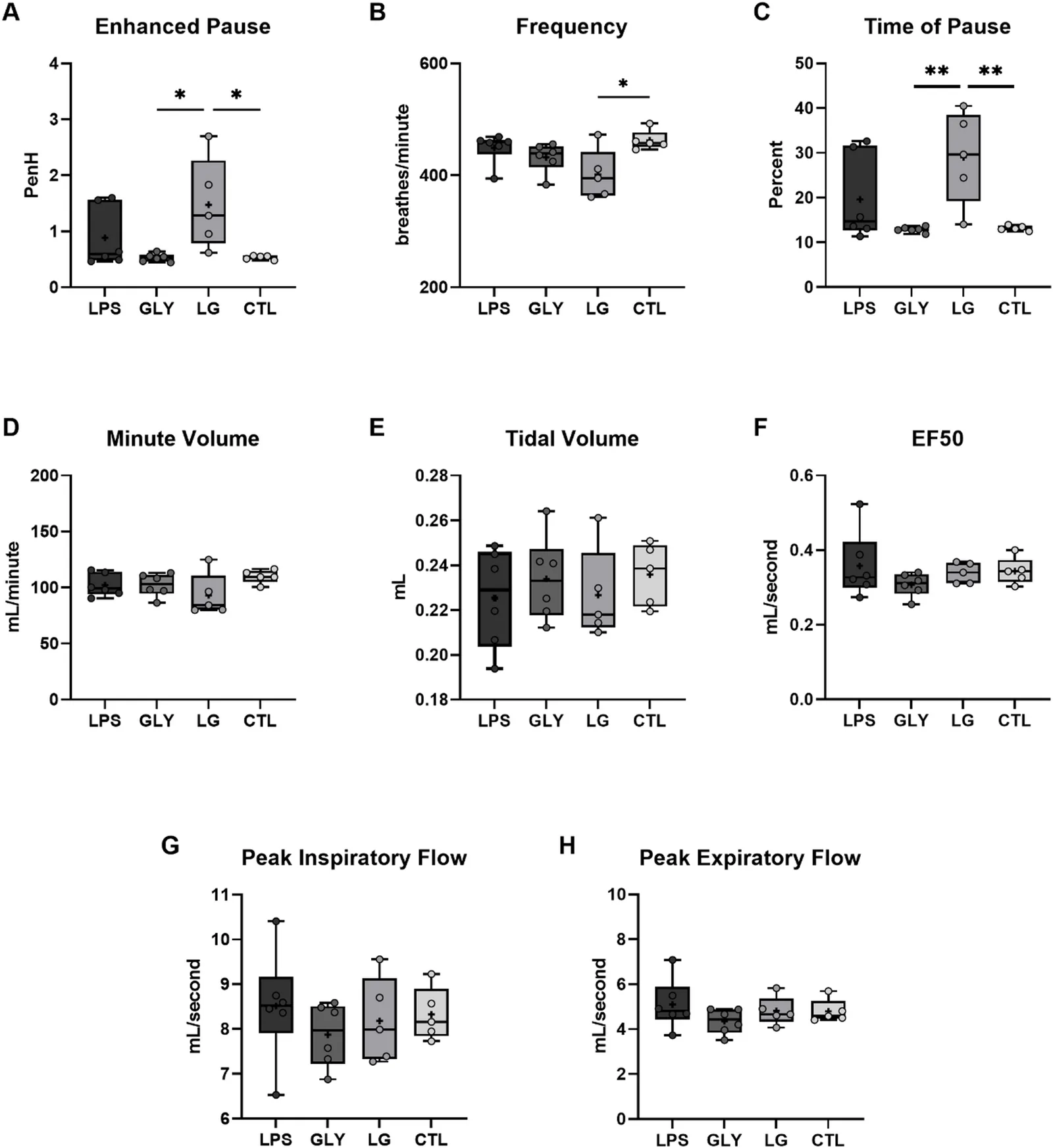
Whole body plethysmography (WBP) data presented as minimum, maximum, median (line), and mean (plus sign) (*n* = 5–6). Mice were exposed to LPS, GLY, LG or CTL for 5-days. WBP data was averaged for 20 min and presented as mean + SD. * indicates significance level of *p* < 0.05.

Enhanced pause (PenH, Fig. 1A) and Time of Pause (Fig. 1C) were significantly higher in the LG group compared to the GLY (*p* = 0.0091 and *p* = 0.0050, respectively) and CTL (*p* = 0.0141 and *p* = 0.0092, respectively) groups, but not significantly different from the LPS group. Average breath frequency was significantly lower in the LG group as compared to the CTL group (*p* = 0.0298, Fig. 1B), but no other differences existed between the groups.

### Hormone analysis

**Fig. 2:**
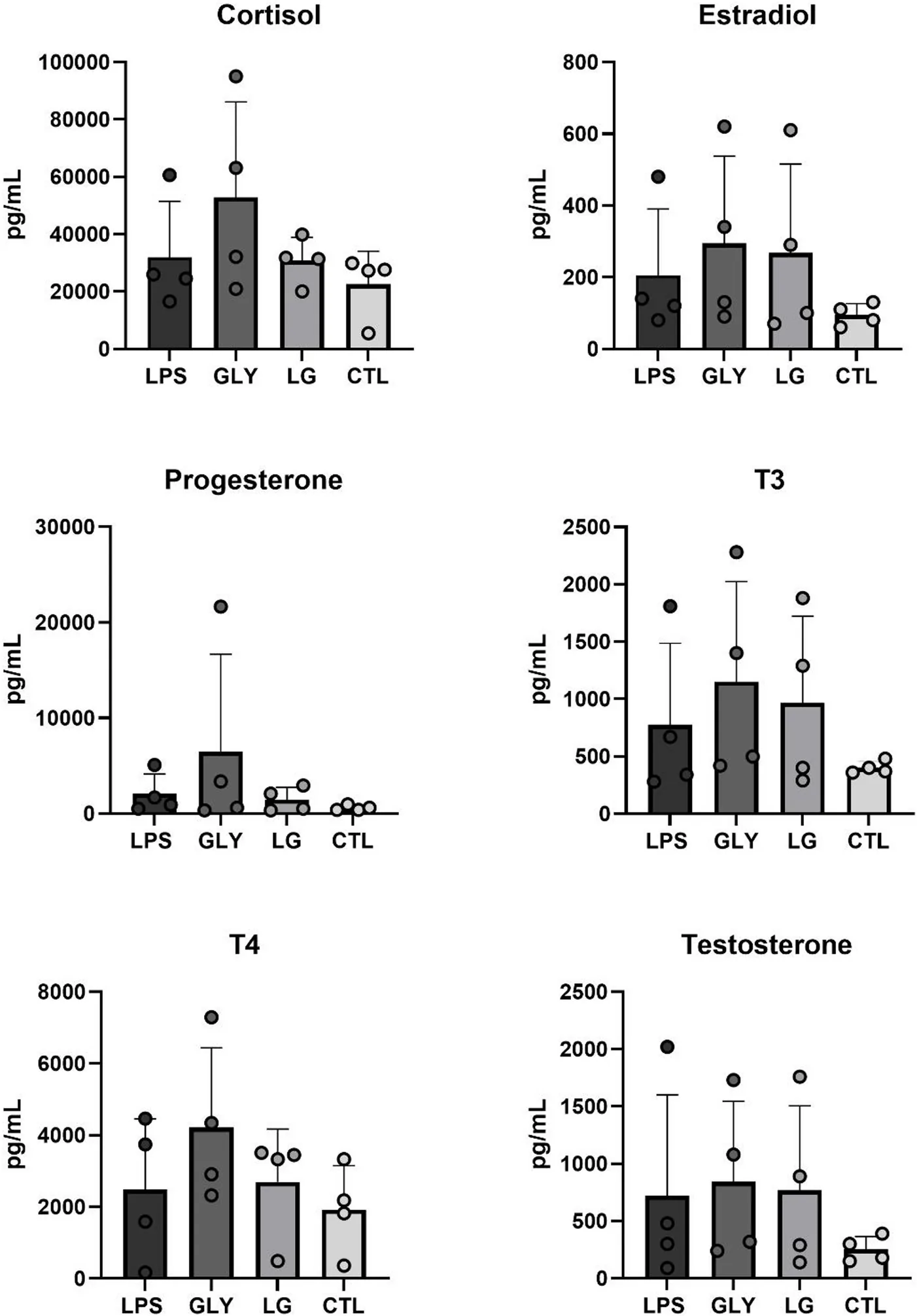
Hormone analysis from mouse blood samples. Mice were exposed to LPS, GLY, LG or CTL for 5-days. Presented as mean + SD (*n* = 4/group). No significant difference was detected between any of the treatment groups.

There were no significant differences between any of the treatment groups for the hormone panel analysis of estradiol, testosterone, cortisol, progesterone, T3, and T4 (Fig. 2).

### Leukocyte counts in bronchoalveolar lavage fluid

**Fig. 3:**
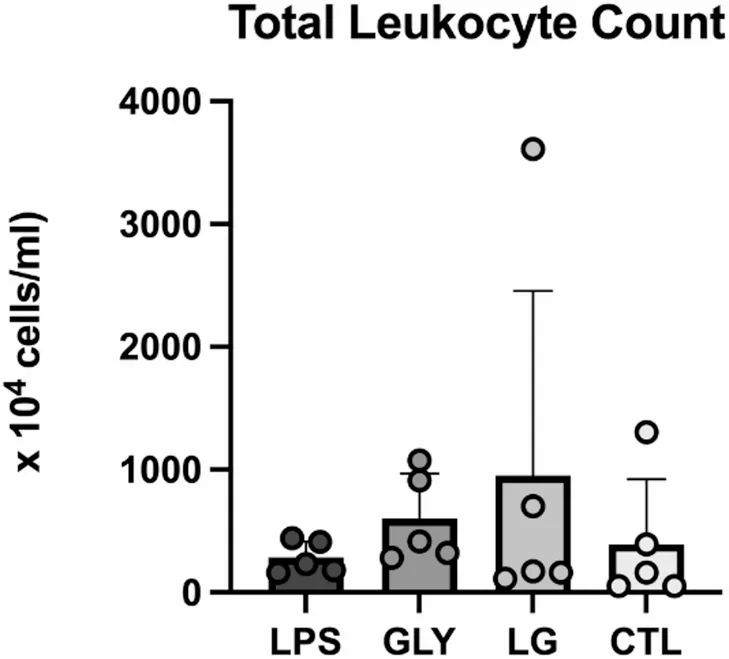
Total leukocyte counts present in bronchoalveolar lavage fluid. Mice were exposed to LPS, GLY, LG or CTL for 5-days. Presented as mean + SD (*n* = 5). No significant difference was detected between any of the treatment groups.

There were no significant differences in total leukocytes between any of the treatment groups (Fig. 3).

### Protein expression in the lungs

**Fig. 4:**
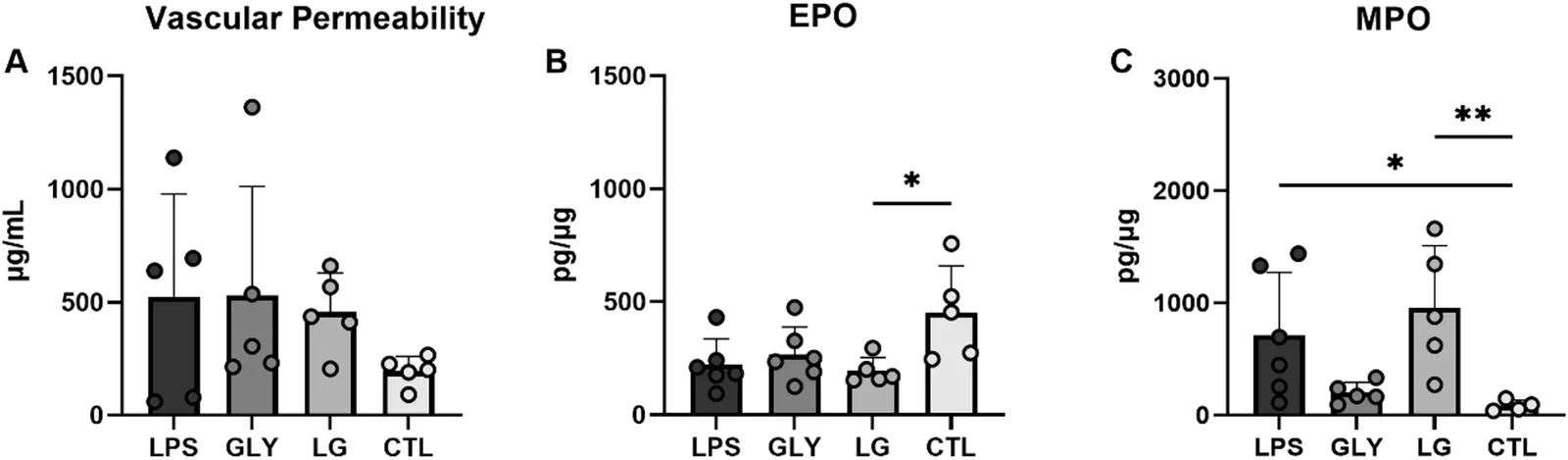
Protein concentration data for vascular permeability (**A**), EPO (**B**), and MPO (**C**). . Mice were exposed to LPS, GLY, LG or CTL for 5-days. Presented as mean + SD (*n* = 5-6). * indicates significance level of *p* < 0.05. ** indicates significance level of *p* < 0.01.

There were no significant differences in vascular permeability between any of the treatment groups (Fig. 4A).

EPO concentration was significantly lower in the LG group as compared to the CTL group (*p* = 0.0352, Fig. 4B).

The MPO concentration was significantly higher in the LPS and LG groups as compared to the MPO concentration of the CTL group (*p* = 0.0466 and *p* = 0.0076 respectively, Fig. 4C). There were no significant differences in either MPO or EPO expression between the LPS and LG groups.

### Lung histology

**Fig. 5:**
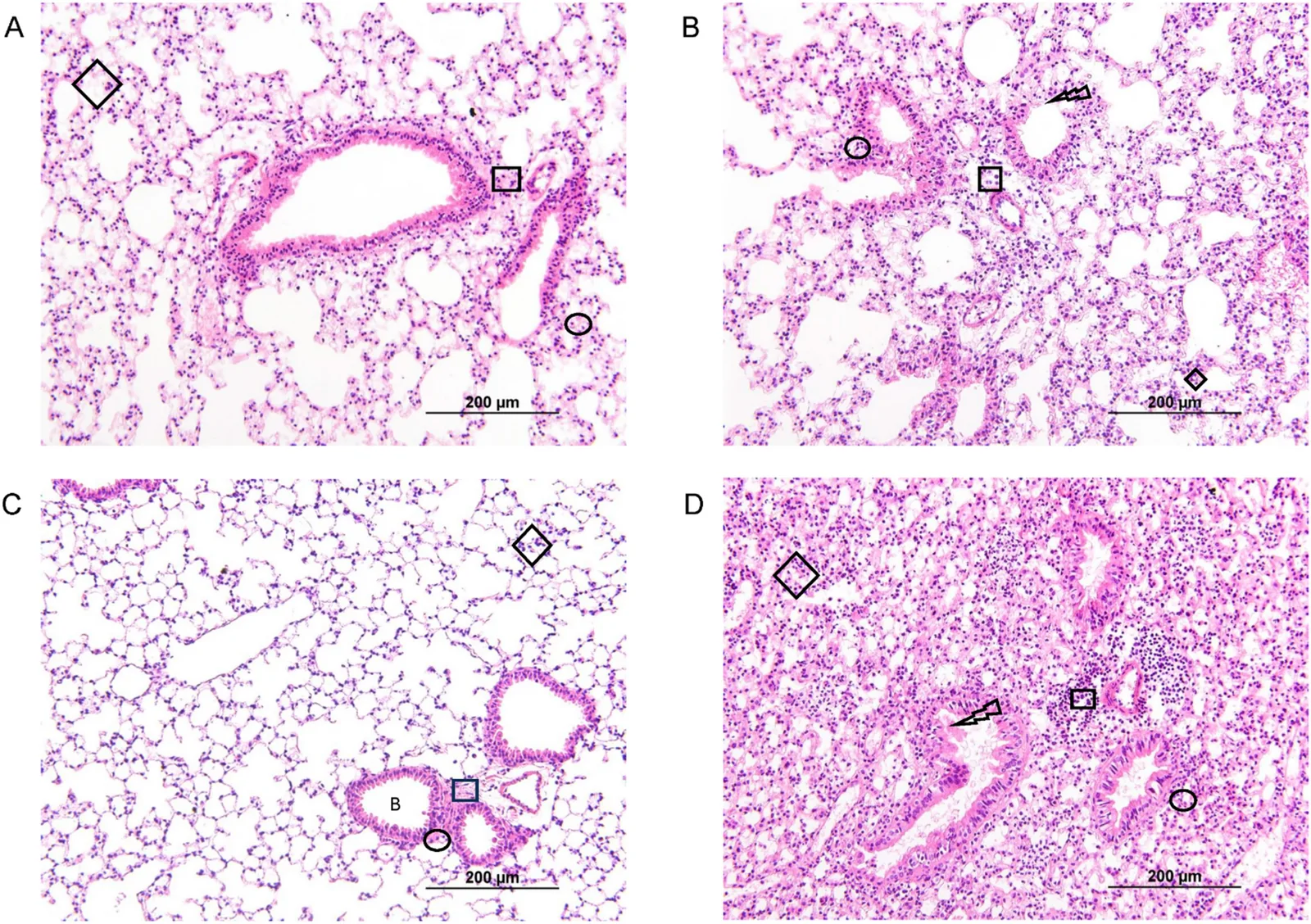
H&E staining of female mice lungs of CTL animals (**A**) or treated with LPS (**B**), GLY (**C**), or LG (**D**) for 5 days. Images show leukocyte infiltration in perivascular (square), peribronchiolar (circle), and alveolar (diamond) regions. Lightning bolt – epithelium disruption. B: Bronchus. Magnification: 40x objective.

Representative images from treatment groups showed leukocyte infiltration in the perivascular, peribronchial, and alveolar regions of the GLY group and LPS group, whereas robust leukocyte infiltration was present in these same regions in the LG treatment group (Fig. 5). Disruption and thickening to the alveolar epithelium were also present in the LPS and LG groups. Increased thickening of the bronchial epithelium was present in the lungs of the LG group.

### ICAM-1 staining

**Fig. 6:**
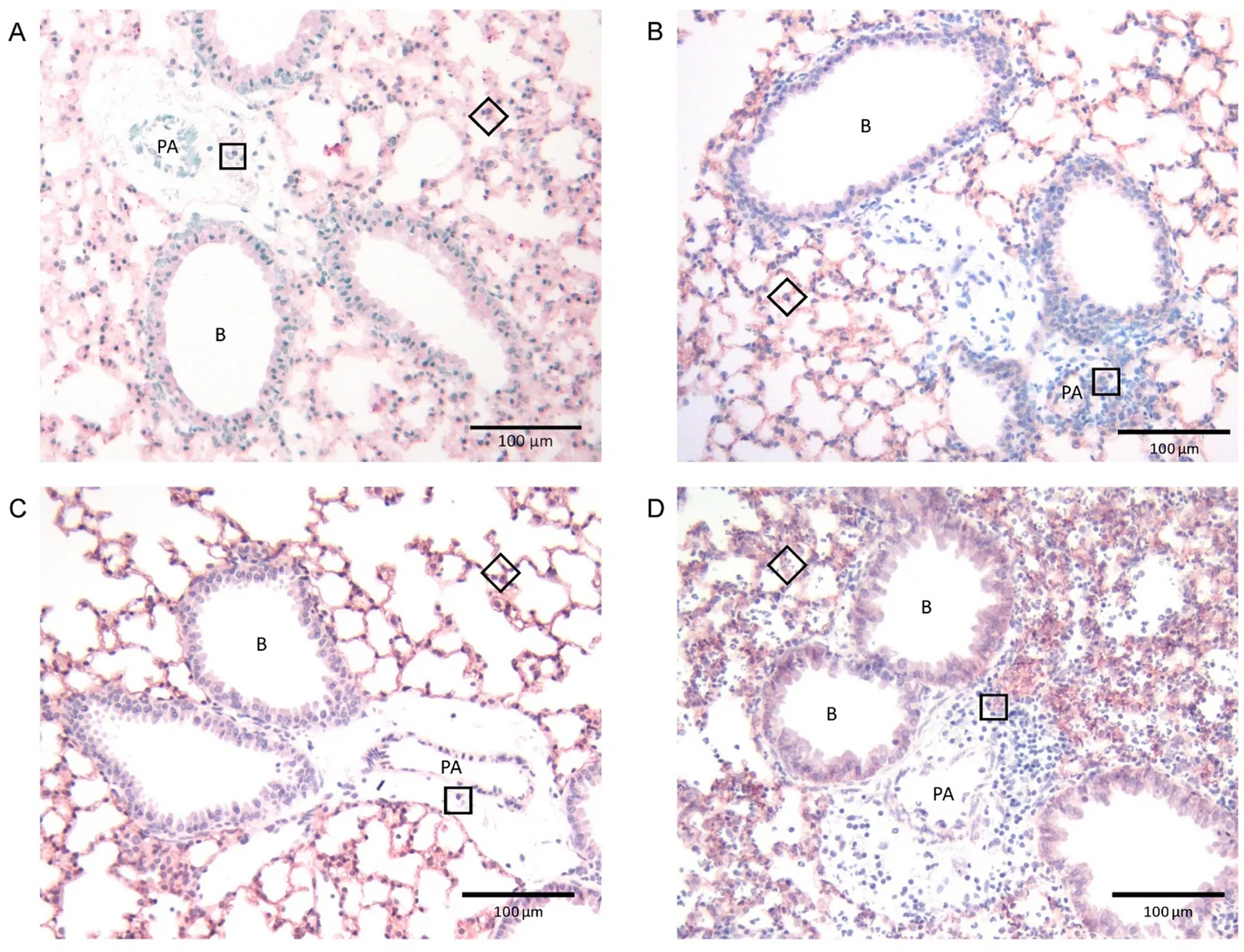
ICAM-1 staining in female mice lungs of CTL animals (**A**), or treated with LPS (**B**), GLY (**C**), or LG (**D**) for 5 days. Darker red staining in the membrane and/or cytoplasm of cells present in the alveolar and perivascular regions of the lung sections are indicative of ICAM-1 positive staining. Images show ICAM-1 staining in perivascular (square) and alveolar (diamond) regions. Magnification: 20x objective; scale bar: 100 μm. B – Bronchus; PA – Pulmonary Artery.

Figure 6 shows representative images of ICAM-1 staining in the alveolar and perivascular regions of the lungs from the treatment groups (Fig. 6). The LPS and LG treatment groups showed more intense ICAM-1 staining in the perivascular region of the lungs as compared to representative sections from the GLY and CTL treatment groups. The perivascular regions of the GLY and CTL treatment groups showed little to no ICAM-1 positive staining. The LPS, GLY, and LG treatment groups showed intense ICAM-1 staining in the alveolar regions of the lung compared to the CTL treatment group.

### Cytokines in BAL

**Fig. 7:**
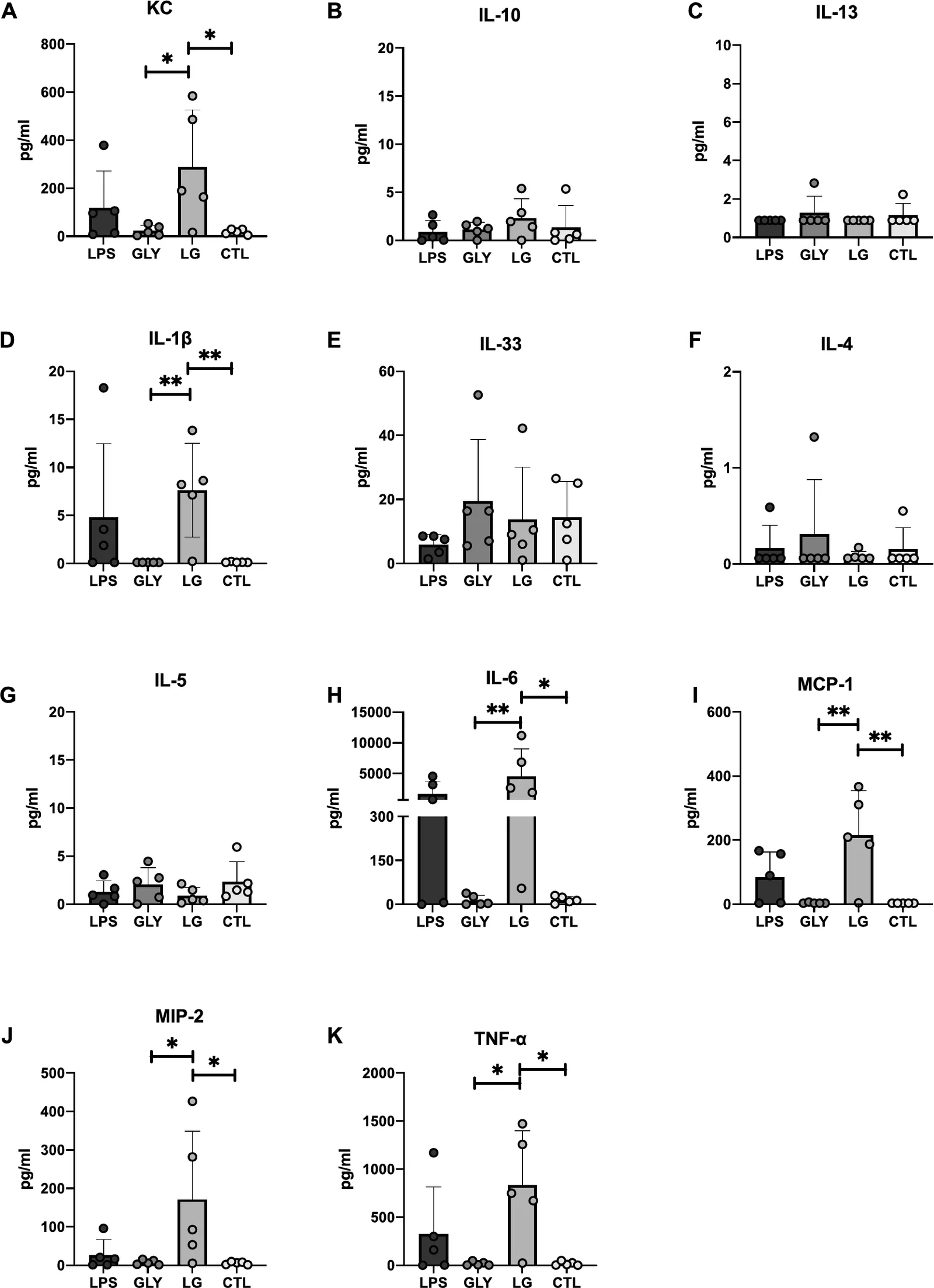
Cytokine levels in bronchoalveolar lavage fluid. Mice were exposed to LPS, GLY, LG or CTL for 5-days. Presented as mean + SD (*n* = 5). * indicates significance level of *p* < 0.05. ** indicates significance level of *p* < 0.01.

The LG treatment group showed a significantly higher expression of KC, IL-1β, IL-6, MCP-1, MIP-2, and TNF-α compared to the GLY and CTL groups (Fig. 7). There was no significant difference between the LG and LPS group for any measured cytokines. There were no significant differences between any of the treatment groups for the expression of IL-10, IL-13, IL-33, IL-4, and IL-5 (Fig. 7).

### ICAM-1, TLR-2, TLR-4, HSPa1a, and TNFAIP3 expression in lungs

**Fig. 8:**
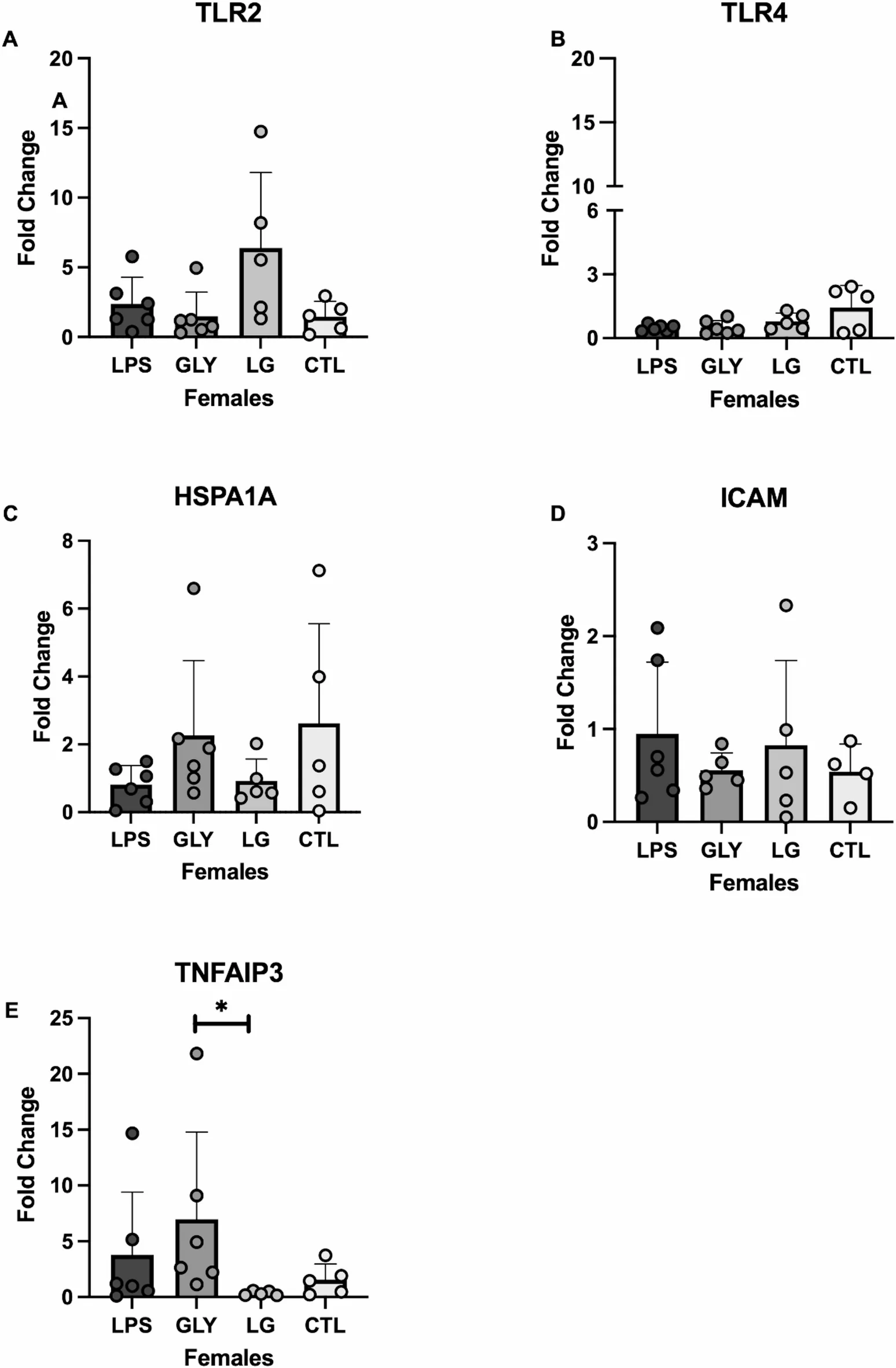
Real time PCR of TLR-2 (**A**), TLR-4 (**B**), HSPA1A (**C**), ICAM-1 (**D**) and TNFAIP3 (**E**) from mice lung tissue presented as fold change. Mice were exposed to LPS, GLY, LG or CTL for 5-days. Presented as mean + SD (*n* = 5–6). * indicates significance level of *p* < 0.05.

The GLY treatment group showed a significantly higher expression of TNFAIP3 compared to the LG treatment group (*p* = 0.0161, Fig. 8E). There was no significant difference between any of the treatment groups for the expression of ICAM-1, TLR-2, TLR-4, and HSPa1a (Fig. 8A-D). There was no significant difference between the LPS and LG groups for any of these markers.

### MIR image analysis

**Fig. 9:**
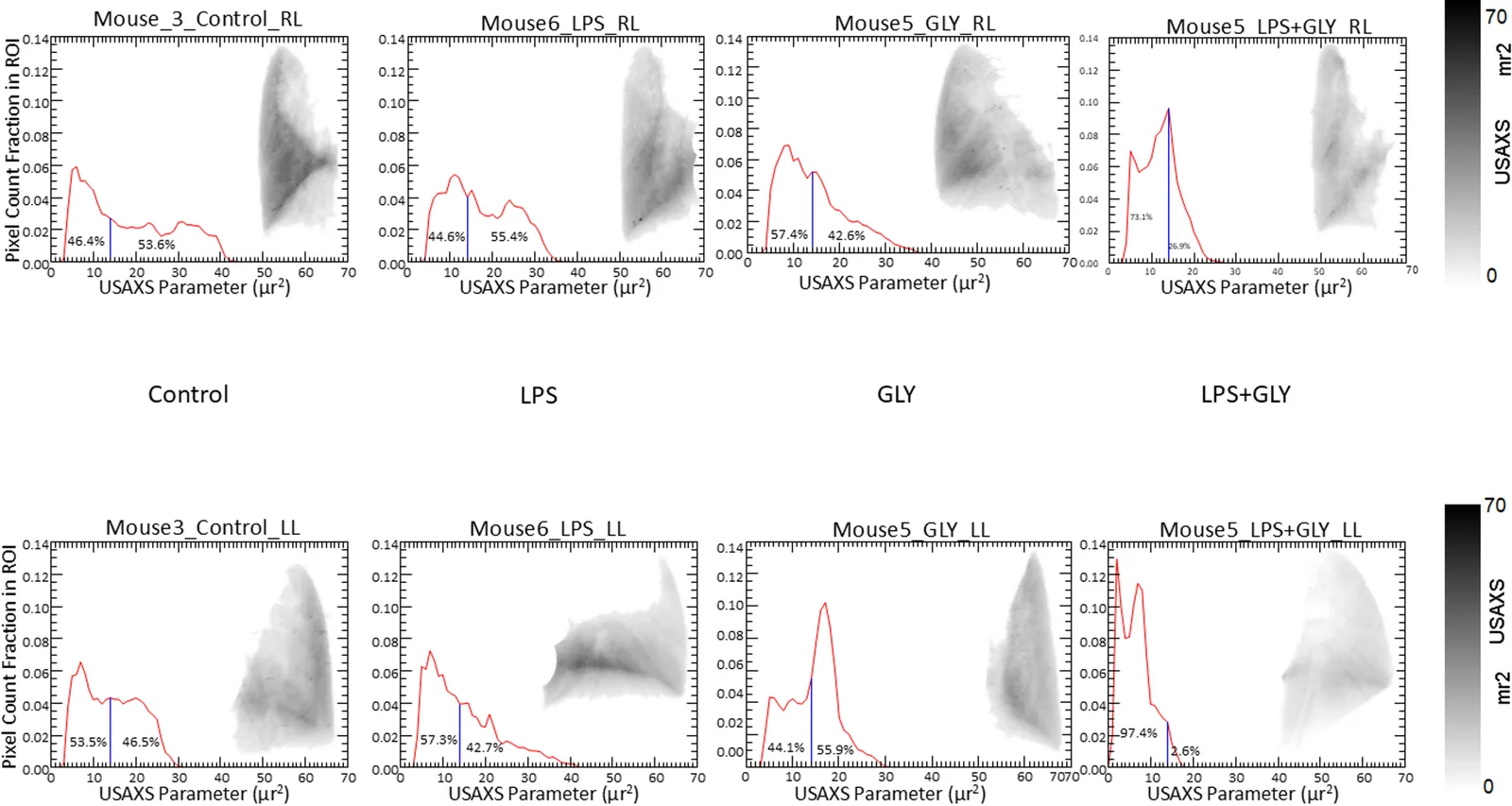
Analysis of Ultra small angle x-ray scatter images of left (L) and right (R) female mice lungs for CTL animals (**A**) or treated with LPS (**B**), GLY (**C**), or LG (**D**) for 5 days. Images are inverted for better visualization of ultra small angle x-ray scatter.

Representative ultra-small-angle X Ray Scatter (USAXS) image analysis show and confirm that lungs possess the maximum number of alveolar air interfaces in the central regions as indicated by black pixels to represent high USAXS parameter. Note the concentric reduction in USAXS parameter along the lung circumference as a result of reduction in the number of alveolar interfaces (Fig. 9). There is a decrease in USAXS values in the LPS and LG animals as compared to the CTL and GLY animals. This is represented by the predominance of white lung regions in the LPS and LG groups lungs.

Ultra-small-angle X Ray Scatter (USAXS) images show that lungs possess the maximum number of alveolar air-tissue interfaces in the central regions to represent high USAXS parameter (> 17 µrad2). Note the concentric reduction in USAXS parameter (< 17 µrad2) along the lung circumference as a result of reduction in the number of alveolar interfaces (Fig. 9). The histograms (Fig. 9) show there is reduced scatter in the LG animal, especially in the left lung (Fig. 9) represented by the increase of pixel count fraction that were below the median of 14 µrad2 in CTL lungs.

**Fig. 10:**
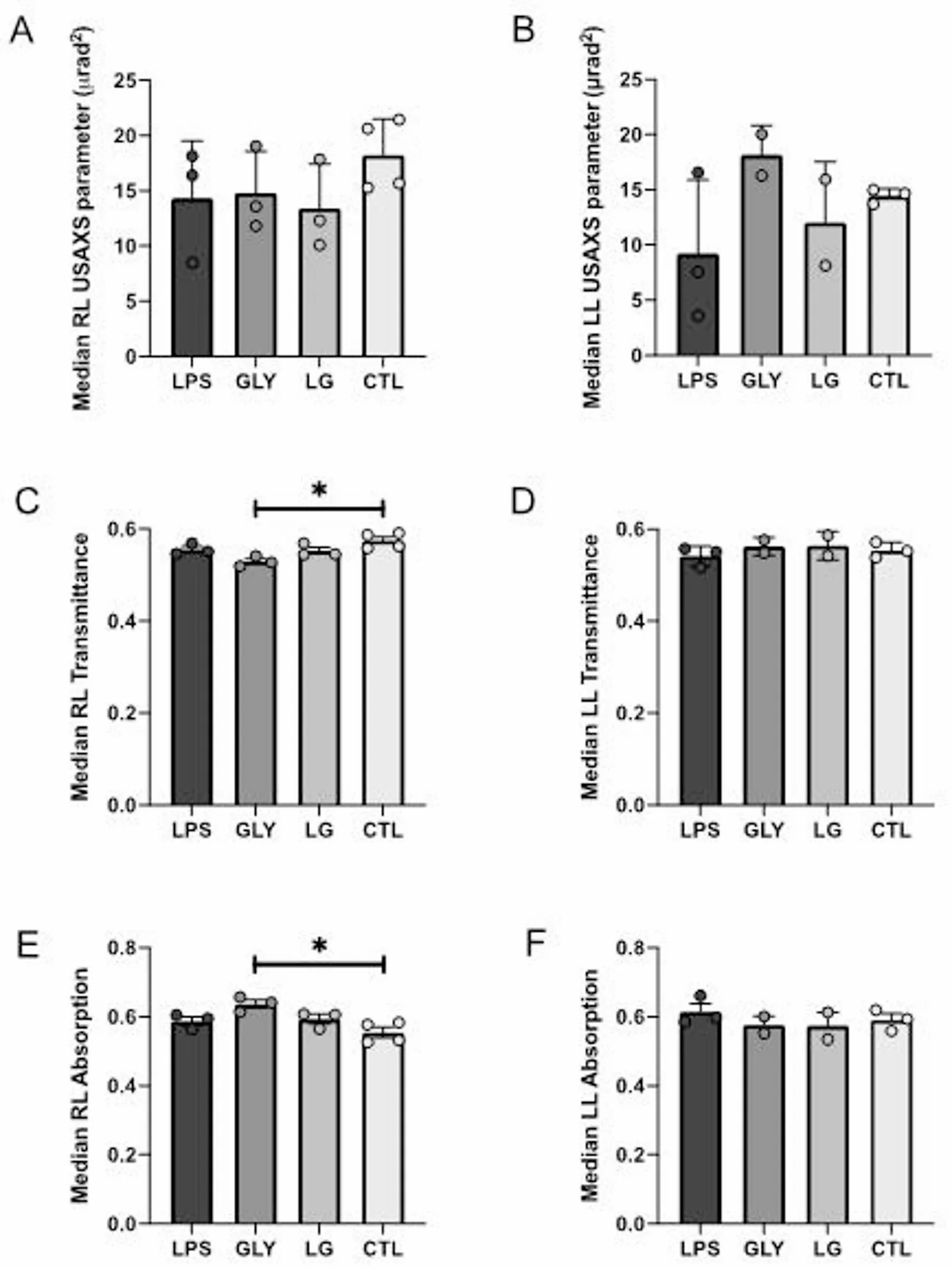
Median lung USAXS squared present in the right lung (**A**) and left lung (**B**). Median lung transmittance in the left (**C**) and right (**D**) lung. Median radiographic absorption in the left (**E**) and right (**F**) lung. Female mice were exposed to LPS, GLY, LG or CTL for 5-days. Presented as mean + SD (*n* = 2–4). * represents *p* < 0.05.

There were no significant differences between any of the treatment groups for median lung USAXS parameter in either the right or left lung lobes. There is a decrease in USAXS values in the LPS (median USAXS of 7.554 µrad2; range 3.59–18.16 µrad2) and LG (median USAXS of 12.04 µrad2; range 8.14–17.86 µrad2) animals compared to the CTL (median USAXS of 14.67 µrad2; range 13.71–21.44 µrad2) and GLY (median USAXS of 18.17 µrad2; range 3.59–16.58 µrad2) animals. This is represented by the predominance of white lung regions in the LPS (57.3% of the pixels in the left and 44.6% of the pixels in the right lung are below 14 µrad2, Fig. 9) and LG (97.4% of the pixels in the left and 73.1% of the pixels in the right lung are below 14 µrad2) lungs (Fig. 9) compared to the CTL (53.5% of the pixels in the left and 46.4% of the pixels in the right lung are below 14 µrad2, Fig. 9) lungs, which is reflected in the USAXS occupied lung pixel fractions for representative lungs (Fig. 9). Interestingly, glyphosate treated left lung displayed predominantly higher USAXS (µrad2) pixel values which are black and occupied 59.9% of the pixels in left lung above 14 µrad2 (Fig. 9). The lung transmittance and radiographic absorption are indicative of the attenuation characteristics. We observed a reduction in right lung transmittance in the GLY group (median transmittance 0.527; range 0.519–0.541) compared to control (median transmittance 0.5745; range 0.558–0.591) group (*p* = 0.0132 for overall differences and *p* < 0.05 for GLY vs. CTL, Fig. 10). There was a simultaneous increase in right lung radiographic absorption in the glyphosate group (median absorption 0.6410; range 0.614–0.657) compared to CTL group (median absorption 0.5545; range 0.526–0.583) (*p* = 0.0123 for overall differences and *p* < 0.05 for GLY vs. CTL, Fig. 10). There weren’t any significant differences observed in the transmittance (Fig. 10) or absorption in GLY treated left lung (Fig. 10).

## Discussion

It is known that the female inflammatory response differs from the male inflammatory response^24^. A sex differential responses to LPS exposures have been described and influenced by various factors including sex hormones wherein estrogen suppressed LPS induced acute lung injury and inhibit the TLR4/TLR2 pathway in female mice, and removal of testosterone decreased immune functions in LPS exposure response^25,26^. Further, histopathological scores for peribronchiolar and perivascular inflammatory cell influx were lower in female LPS-treated mice^27^. While female workers are prevalent in the agriculture industry, there is a scarcity of research on the female inflammatory response to agricultural exposures^24^. This is the first study to evaluate the lung response of female mice exposed to a combination of LPS and GLY, common agricultural exposures, and to use live animal synchrotron imaging to support the findings. The results show that in female mice there is an inflammatory response to LPS, and the addition of GLY has an impact on some inflammatory parameters, but does not result in a robust alteration to inflammatory effects after 5 days of exposure. However, the results do reveal differences in the lungs of the LG mice as compared to the other exposure groups both in the histology and lung injury imaging.

Whole body plethysmography (WBP) is a non-invasive technique to evaluate respiratory parameters. Enhanced pause, or PenH, is one such parameter and is considered to be a marker for airway resistance in the lungs. Airway resistance is a well-known response to LPS exposure in female mice^28^. The WBP results show mice that received LG had higher PenH as compared to the GLY and CTL groups, but not different from the LPS group. LPS administration has been shown to increase breathing frequency^29^. The average breath frequency was lower in the LG group, indicating the difficulty in maintaining a normal rhythm of breath. Previous studies have also observed decreases in respiratory function following LPS exposure at a dose similar to the one used in our study in both male and female mice^27^. Our findings reinforce that a low dose exposure to LPS can inhibit respiratory function and the addition of GLY further compounds these respiratory effects.

The results reveal that myeloperoxidase expression is significantly higher in the LPS and the LG treated mice. It has well been established that exposure to LPS leads to the recruitment and activation of neutrophils^30^. Myeloperoxidase is a surrogate marker for the presence of neutrophils; however, TLR-4, a ligand involved in neutrophil recruitment^31^, was not upregulated in these treatment groups. Given that these results were after 5 days, it is likely that the adaptive immune response is occurring, as the female inflammatory system is known to quickly respond to LPS^32^.

While EPO, IL-4 and IL-5 are lower, they are not significantly different from the other exposure groups after 5 days. The inflammatory mediator results show that female mice exposed to the combination of LG could not be differentiated from mice exposed to LPS after 5 days of low dose exposure. However, this can be expected given that in female mice inflammatory adaptation has been shown as quickly as 36 h after exposure^32^, the exposures here were five-day exposures, and therefore inflammatory adaptation is likely already occurring, but needs to be confirmed with earlier time-series studies. Further, the TLR-2 expression is of interest. TLR-2 has been shown to be an important pattern recognition receptor (PRR) in asthma-like inflammatory responses^33^. While TLR-2 is not significantly different between the groups, it would be interesting to look at the effect at an earlier time point to better establish any potential asthma-like inflammatory responses that may have begun adapting in the female mice at a time point earlier than 5 days. TNFAIP3 is an important anti-inflammatory protein present in endothelial cells^34^. The GLY mice displayed higher levels of TNFAIP3 compared to the LG group, indicating the GLY mice may be regulating their immune response. To the best of our knowledge, the interaction between TNFAIP3 and glyphosate has not been studied, but these results suggest it may be worth exploring.

The H&E, immunohistochemistry, and synchrotron imaging results add additional support of the potentiation of respiratory response from a combined LG exposure as compared to LPS alone. Representative images from the LG treated lung tissue shows robust leukocyte infiltration, thickening of the alveolar epithelium and bronchial epithelium, and ICAM-1 staining in the perivascular region; that appear more robust than in the LPS only images. The ICAM-1 immunohistochemistry staining and ICAM-1 PCR expression results do not show a strong response overall, however, the LPS and LG treatment groups show a slightly stronger response compared to the GLY and CTL groups, although not significantly. These results differ from the work of Pandher and colleagues who found that in male mice the same concentration of LG in a 5-day exposure in male mice led to an asthma like phenotype that was distinguished by higher neutrophil numbers, higher expression of TLR-2, and changes in lung architecture^7^.

Based on the various inflammatory mediators measured, the systemic effects of the combined exposure do not differ from the LPS alone exposure group after a 5-day exposure in female mice; however, the increased thickening of the bronchial epithelium evident in the histology results supports that the lung architecture sustained effects from the combined exposure that were different from the LPS exposure group.

Hormone levels in female mice are known to contribute to regulating the inflammatory response^26^. In our study there was no difference in hormone expression between any of the treatment groups. Exposure to some pesticides has been associated with hormone disruption^35,36^. There were no differences between any of the treatment groups for the hormone panel analysis of estradiol, testosterone, cortisol, progesterone, T3, and T4 (Fig. 2). As hormone levels are known to fluctuate throughout lifetime, especially in females, another study evaluating if the results observed in our 6–8-week-old female mice changes during different time periods (such as during pregnancy or post menopause) would add considerable understanding to this area of research. The MIR results from the synchrotron imaging revealed differences between the LG exposed images compared to the other treatment groups. In the width or USAXS images, scatter is related to the number of boundaries or interfaces of air and tissue^16^. In a normal lung, there are more interfaces, or alveoli, present in the middle of the lung and these alveoli or interfaces gradually decrease towards the periphery. In a compromised lung, there will be less scatter visualized in the centre of the lung indicating fluid presence and disruption of the alveoli. Both the LPS and the LG treated lungs exhibit less scatter represented by white areas in the inverted USAXS images, especially in the apical regions of the lung. The white portions in the inverted USAXS images of the lung, where lung tissue is expected to be, are indicative of loss of air-tissue interfaces due to fluid in the lung region or a compromised lung region. For X-ray interactions, tissue and fluid will behave the same and not result in x-ray USAXS. Although total scatter in the lung can be quantified using the measurement “rawintdens” of the USAXS parameter (µrad^2^), we did not include it in our current analysis owing to non-uniformity in the lung regions imaged across mice. We ascribe two main reasons for the high variation in the USAXS parameter. Firstly, not all animals were imaged uniformly. Some of the mice lungs were imaged partially leading to variation in the region that was accounted for in the analysis. MIR is direction sensitive; during analysis we realized that some mice were oriented at an angle compared to others which were in a vertical orientation, which attributed to different distribution patterns of USAXS. Secondly, the beam and detector displayed non-uniform flat and dark normalized images, which is a technical issue with the MIR image acquisition set-up.

An interesting observation from the MIR analysis was the reduction in lung transmittance and an increase in absorption in GLY treated mice compared to CTL group as both these parameters are related. This observation suggests that GLY either induces fluid accumulation or tissue fibrosis which warrants carefully planned future studies in order to confirm the relevance of these observations. Additionally, the USAXS histograms also suggest abnormal fluid accumulation or cellular infiltration, as there was abnormal scatter present in the GLY treated mice. However, there is an insufficient sample size to deduce these changes from our current histological and plethysmography results.

Kaur et al. observed statistically significant changes in LPS treated mice lungs across various parameters of the width/USAXS images^17^. Although the influence of LPS and LG is visible in the actual images, differences between the two groups did not come through in the statistical analysis. However, this current study used a small sample size and variable mouse positioning which led to increased variability across measurements. Additionally, the decrease in scatter for some animals was varied between left and right lung lobes, and variation was observed even regionally within the same lobe (i.e. apex vs. base of lung). In future studies, as the procedural processes improve, we are confident these qualitative changes observed will translate into quantitative changes that are statistically significant and biologically relevant.

There are several limitations to these results. The sample size of 5 per treatment group was used based on results from prior studies showing this was the smallest number we could use to be able to discern significant differences between treatment groups while still minimizing the number of animals used in our study. Increasing the sample size of each treatment group would increase the power and robustness of our statistical analysis to be able to discern if inflammatory differences existed between the groups. These results are from a 5-day exposure. An evaluation at an earlier timepoint may reveal different inflammatory patterns between the groups. The lack of tracking the estrous cycle of the female mice is a further limitation of the study. It is well established that sex hormone fluctuations in the estrous cycle can significantly regulate TLR-4 mediated signaling, NF-kB activation and the subsequent inflammatory responses^37^. A difficulty we encountered was that differential cell counts (DLC) could not be performed due to issues with our protocol. As the results from our total cell count did not show any differences between treatment groups, the DLCs may have provided valuable additional insights.

The strengths of this study are that the concentrations of LPS and glyphosate used are based on those found in real world exposures. The concentration of glyphosate used is one that has been used in previous studies in the same strain of mouse. The study incorporated a number of different respiratory assessment parameters important to furthering our understanding of lung injury from these exposures. The MIR component of this paper was a pilot study to orient the research team to the BMIT-BM beamline at the CLS and refine the MIR technique. These MIR results are a new and unique contribution to this area of research. There was a steep learning curve associated with the MIR component. Based on this pilot work, multiple steps have been streamlined that will be invaluable moving forward with similar experiments including refining the experimental procedure and productivity during the actual beamline shifts, and the stacking and analysis of the field of view images acquired. Now that these processes have been developed, future studies can benefit.

## Conclusion

This study is the first to report the impact of two common agricultural respiratory contaminants, LPS and glyphosate, on the lungs of female mice following 5 days of exposure using multiple techniques to evaluate lung effects including whole body plethysmography, multiple image radiography, and histology. Female mice, exposed to LPS and glyphosate for 5 days showed higher levels of inflammatory mediators compared to control animals, or those treated with only glyphosate. While the inflammatory mediator results observed in the combined LPS and glyphosate treatment group were not significantly different from the LPS exposure group, the histology and multiple image-radiography results reveal lung effects that are more robust in the combined LPS and glyphosate treated animals. Taken together, these results reveal that female mice exposed to the combination of LPS and glyphosate displayed physiological and structural lung effects that were different from mice exposed to LPS or glyphosate alone.

## Data Availability

The datasets used and/or analyzed during the current study are available from the corresponding author upon reasonable request.
